# Implementation and effectiveness of a food education intervention to promote plant-based foods: a partially cluster-randomized controlled trial

**DOI:** 10.1186/s12966-026-01924-x

**Published:** 2026-04-22

**Authors:** Henna Vepsäläinen, Satu Kinnunen, Sari Niinistö, Leena Forma, Suvi T. Itkonen, Tuuli E. Korhonen, Liisa Korkalo, Heli Kuusipalo, Jelena Meinilä, Kaija Nissinen, Susanna Raulio, Ros Sambell, Mari Åkerlund, Suvi M. Virtanen, Maijaliisa Erkkola

**Affiliations:** 1 Department of Food and Nutrition, University of Helsinki, PO Box 66, Helsinki, 00014 Finland; 2 Department of Public Health, Finnish Institute for Health and Welfare, PO Box 30, Helsinki, 00271 Finland; 3 Faculty of Social Sciences, Unit of Health Sciences, Tampere University, Arvo Ylpön katu 34, Tampere, 33520 Finland; 4 Department of Health and Social Management, University of Eastern Finland, PO Box 1627, Kuopio, 70211 Finland; 5 Seinäjoki University of Applied Sciences, PO Box 412, Seinäjoki, 60101 Finland; 6 School of Medical Sciences, Nutrition & Health Innovation Research Institute, Edith Cowan University, 270 Joondalup Drive, Perth, WA 6027 Australia; 7 Tampere University Hospital, Wellbeing Services County of Pirkanmaa, PO Box 272, Tampere, 33101 Finland; 8 Centre for Child Health Research, Tampere University and Tampere University Hospital, Tampere, Finland

**Keywords:** Planetary health, Sustainability, Daycare, Early childhood education and care, Pre-schoolers, Nutrition education, Pulses, Legumes, Implementation degree

## Abstract

**Background:**

Legumes can substitute for meat and are thus a particularly important food group for environmental sustainability. As the use of legumes among Finnish children is marginal, there is potential for a substantial increase in their consumption. However, food education delivered through early childhood education and care (ECEC) settings has rarely focused specifically on environmentally sustainable foods, such as legumes. This study describes the implementation and effectiveness of an ECEC-delivered food education intervention within the FoodStep study.

**Methods:**

The partially cluster-randomized FoodStep intervention aimed to increase the supply of vegetables, legumes, fruits, berries, and sustainable fish while reducing the provision of other animal-based foods in the intervention ECEC centres. The control centres maintained their regular menus. Food education materials and training were included to support the acceptability of the menu changes. Based on implemented food education activities reported by the ECEC professionals during the 10-month intervention, the ECEC centres were classified into high-intensity intervention (*n* = 5), low-intensity intervention (*n* = 6), and control group (*n* = 12). Parents of the participating 3- to 5-year-old children (*n* = 86) reported their child’s willingness to taste foods using a five-point scale. Food consumption was assessed using two separate food frequency questionnaires: (1) foods consumed outside ECEC and (2) foods consumed in ECEC. We used linear mixed models to examine the effectiveness of the food education intervention in increasing food acceptance and consumption.

**Results:**

Commonly implemented food education activities were sensory food education, books and drama, gardening, and crafting. Compared with the control group, the children in the high-intensity intervention group showed higher acceptance of (β = 4.27, 95% CI − 0.03 to 8.57, *p* = 0.06), and consumed more, legumes at the follow-up (β = 1.18, 95% CI 0.31 to 2.06, *p* = 0.01). No statistically significant associations were observed for the other food groups.

**Conclusions:**

ECEC-delivered food education interventions accompanied by accessible materials and activities can help to promote sustainable diets in children by increasing the acceptability and consumption of environmentally sustainable foods, such as legumes. To encourage sustainable diets, menu modifications should be supplemented with pedagogic food education activities in ECEC centres.

**Trial registration:**

ClinicalTrials.gov (NCT05249946) November 29, 2021.

## Background

 The current food systems are under scrutiny for their contribution to greenhouse gas emissions, their impact on biodiversity, and use of freshwater. These critiques frequently highlight the need for dietary shifts towards a sustainable diet rich in whole grains, legumes, vegetables, fruits, and sustainably produced fish, with moderate amounts of other animal-based products [1, 2]. This is broadly in line with the current Finnish National Nutrition Recommendations [3], but significant environmental benefits can only be achieved through a stronger emphasis on plant-based diets [4]. Legumes are an especially important food group, because they can substitute for meat, thus reducing environmental impacts. Although legumes are recommended for children from the stage of complementary feeding, data on legume consumption in children are limited [5]. However, consumption seems to be below recommendations especially in high-income countries [5]. In a Finnish study of 3- to 6-year-olds, legume consumption was around 5 g/d [6], which corresponds to approximately 10% of the target set by the EAT–*Lancet* Commission [1].

Dietary challenges related to environmental impact and health overlap in the diets of Finnish children. Animal-based foods are a significant source of saturated fatty acids [7, 8], and partial replacement of meat and fat-containing dairy products with plant-based products would improve fat quality in the diet. Higher consumption of whole-grain products and legumes would increase the intakes of dietary fibre and folate. A shift towards a more plant-based diet would improve the nutritional quality, and potentially reduce the environmental impact, of children’s diets [9]. As eating habits adopted in childhood typically continue into adulthood [10, 11], promoting healthy and sustainable diets is especially important in childhood.

Early childhood education and care (ECEC) is an ideal, yet probably underutilized, arena for increasing the share of plant-based foods eaten across socioeconomic subgroups, thus promoting social sustainability. Food education, defined as all pedagogic activities, counselling, guidance, and communication related to food and eating, can support the acceptability of unfamiliar, healthy, and sustainable ingredients, foods, and dishes in ECEC. Multiple studies have found positive or borderline positive effects between ECEC-delivered food education activities and children’s willingness to taste [12–15] or consume fruits and vegetables [16–23]. The most frequently reported components in these interventions have been sensory play activities or child participation in cooking [12–17, 20, 22], transmission of information to parents either through lessons or in written form [17, 18, 21, 22], and pedagogic, lesson-like activities for children [17, 18, 20, 21]. Some interventions supported food education activities by increasing taste exposure [14, 20, 22]. However, the studies have not investigated which of the components were most frequently used. Thus, it remains unknown which types of food education materials are most feasible in the ECEC environment.

While it is important to identify strategies to increase the consumption of fruits and vegetables, reducing the environmental impact of food requires a particular focus on promoting the use of foods, such as legumes, that can partially substitute for meat. Partly because of their sensory properties, such as bitterness [24], legumes are often disliked in the adult population [25] and have a relatively small role in Finnish food culture, which is why it would be important to familiarize children with them in ECEC. Only a few studies have examined the effects of ECEC-based food education initiatives on willingness to taste legumes or legume consumption. A study conducted on a sample of 523 US children found that children exposed to a food education intervention were more likely to consume edamame (young soybeans) at follow-up [26]. Similarly, a UK study showed a tendency towards increased green bean consumption in 2- to 4-year-olds following a 30-day intervention [27]. However, the study only included 20 participants, and no statistical analyses were conducted. In a US study, willingness to try snap peas increased among children exposed to a food education intervention, whereas no difference was detected among children in the control group [28]. One US study of 4- to 6-year-olds was unable to observe any intervention effects on parent-reported green bean consumption following a teacher-led food education intervention [29]. Taken together, some evidence suggests that food education can increase legume consumption, but because of the small number of studies, inconsistent results, and differing and inadequate study designs, no firm conclusions can be drawn.

To summarize, evidence shows that food education delivered in ECEC settings may have a beneficial effect on children’s diet, especially fruit and vegetable consumption. However, there is a lack of studies focusing on increasing the acceptability and consumption of environmentally sustainable foods, such as legumes. Further, more information is needed on which food education materials and activities are most feasible in the ECEC environment. To fill these gaps, we explored (1) how the FoodStep food education intervention was implemented and (2) the effectiveness of the intervention in increasing sustainable food acceptance and consumption among 3- to 5-year-olds. An additional aim was to investigate the associations between food acceptance and consumption measures. The results generated in this study are urgently needed to support development of easily implementable, sustained societal-level food education practices that can foster more plant-based diets in a socially sustainable way.

## Methods

### The FoodStep intervention

The partially cluster-randomized FoodStep trial, preregistered at ClinicalTrials.gov (NCT05249946), aimed to support healthy diets for children and reduce the climate impact of the ECEC food system. The preregistered primary outcomes are reported in separate manuscripts (e.g. [30]). To provide a more comprehensive understanding of the effectiveness of the FoodStep intervention, this article reports implementation- and feasibility-related data and the effectiveness of the food education component within the intervention (secondary findings). The study was conducted in one municipality in southern Finland (Päijät-Häme) and three municipalities in western Finland (South Ostrobothnia), from which a total of 23 ECEC centres (15 in Päijät-Häme and 8 in South Ostrobothnia) agreed to participate. The ECEC centres from Päijät-Häme were randomized (simple random allocation) within four socio-economic strata into the intervention and control arms by the principal investigators in 2/2022. In South Ostrobothnia, randomization was not possible because of logistic reasons and limited kitchen resources, which prevented certain ECEC centres from acting as intervention-arm centres. A total of 11 ECEC centres were allocated to the intervention arm, whereas 12 centres were grouped into the control arm. The total number of children reached by the study was 1221, of which 643 (53%) attended the intervention and 578 (47%) the control ECEC centres.

The intervention consisted of (1) co-creation workshops designed to engage all relevant interest-holders (e.g. decision makers and food service and ECEC professionals); (2) menu modifications, including recipe development in collaboration with the students from the Seinäjoki University of Applied Sciences; and (3) ECEC-delivered food education to support the menu changes and increase the acceptability of the modified menus. The menu modifications aimed to increase the supply of vegetables, fruits, berries, legumes, and environmentally sustainable fish from nearby areas in the ECEC centres as well as to reduce red meat and milk consumption to recommended levels [31]. Due to the nature of the intervention (co-creation, modified menus, and active food education targeting the primary outcomes), neither party was blinded to intervention/control arm allocation. The duration of the intervention was 10 months (March to December 2022). Menu compliance was monitored over the intervention period through regular communication (for more detail, see [30, 32]). The control ECEC centres did not participate in the co-creation workshops, maintained their regular menus, and did not receive any food education support. The intervention was approved by Ethics Committee I of the Helsinki and Uusimaa Hospital District (1553/2021, 23 June 2021). Written informed consent was obtained from a legal guardian (later referred to as a parent) of each participating child.

### The food education intervention

The food education intervention was co-created by experts in food education, nutrition science, and children’s food behaviour, as well as professionals in the fields of ECEC and food services. The co-creation was organized through a workshop aimed at professionals (*n* = 27) of the intervention ECEC centres, focusing on introducing the food education themes and implementing them into food education practices. The co-creation has been described in more detail in a separate manuscript [32]. The co-developed food education intervention consisted of four intervention periods, each having both a food-related and educational topic. The researchers compiled a large number of food education materials, both existing and specifically created for the intervention ECEC centres to be used in their food education activities. All topics, their timings, and materials provided are presented in Table 1.

**Table 1:** Topics and related materials in the FoodStep food education intervention (2022)

Food-related and educational topic	Information materials for the early educators			Food education materials to be used with the children			Training provided for the early educators
		Printed	Online		Printed	Online	
1: Legumes & role modelling	Food education planning form	x		Legume bingo	x		Training on the study aims and role modelling
March–April	Fact sheet about legumes	x		The bean champion game	x		
	Tips about role modelling	x		Diamonds of success		x	
	Blog post about role modelling		x	YAMMY card: legumes		x	
				Mole’s Veggie Adventures mobile application		x	
2: Domestic fish & food waste	Food education planning form	x		Fish bingo	x		
May–June	Fact sheet about fish	x		Diamonds of success	x		
	Tips about food waste	x		Fish recipes	x		
	Crowd-sourced ideas for food education		x	Fish cards, how to identify fishes	x		
	Blog post about fish		x	YAMMY card: fish	x		
	Blog post about food waste		x	Food waste leaflet	x		
				Fish colouring images		x	
3: Self-regulation & berries	Food education planning form	x		Berry bingo	x		Online training on self-regulation
August–September	Fact sheet about berries	x		Diamonds of success	x		
	Tips about self-regulation	x		Kitchen feedback form	x		
	Mindful eating guide for teachers	x		Berry cards, how to identify berries	x		
	Mindful eating handbook		x	Mindful eating poster	x		
	Blog post about self-regulation		x	YAMMY card: berries		x	
	Blog post about berries		x				
4: Encouragement & cabbages	Food education planning form	x		Cabbage bingo	x		
October–November	Fact sheet about cabbages	x		Vegetable wheel	x		
	Tips about encouragement	x		Diamonds of success	x		
	Blog post about encouragement		x	Sensory track	x		
	Blog post about cabbages		x	Cabbage and legume cards	x		
				YAMMY card: cabbages	x		
				List of books with food education contents		x	

All printed materials were also provided as online materials. The online material bank also contained links to other pages listing food education activities

To launch the food education intervention, we organized a 3-hour training session in collaboration with the Finnish Society for Food Education, Ruukku, in each of the intervention ECEC centres. This training session introduced the study in detail and focused on the first topics of the intervention: role modelling and legumes. To maintain the interest of ECEC professionals after the summer holidays, additional online training on children’s eating-related self-regulation and its association with food consumption was given approximately five months later. The intervention-arm ECEC professionals were instructed to organize at least one 20-minute food education session per week. They could use the materials provided by the research team but were also encouraged to search for more materials and select topics and materials they found most suitable for their group. After the intervention period, the control group received a 2-hour training session following the outlines of the intervention training as well as access to all the food education materials. To increase engagement, we also set up social media accounts in Facebook, Instagram, and Twitter (currently known as X). On the study’s website, we published seven blog posts and 12 recipes that closely aligned with the topics of the food education intervention. Social media was used to increase their visibility. A leaflet briefly explaining the importance of food education and inviting parents to follow the study’s social media channels was sent to families through the intervention ECEC centres.

### Assessing the intervention implementation

A total of 36 ECEC groups (62% of the 58 groups that were sent the intervention material) from the 11 intervention-arm ECEC centres journalled their food education sessions using a pen-and-paper food education diary. The diary included information on the dates of the sessions as well as materials used and topics covered. The ECEC professionals also reported whether they had read specific blog posts related to the topics of the intervention and whether any ECEC professional in their group followed the study’s Facebook, Instagram, or Twitter accounts (‘none/one/two or more of the ECEC professionals in the group has read or followed’).

To assess how the intervention-arm ECEC centres had implemented food education activities, we created a measure for the degree of implementation (DOI). The DOI was calculated based on the information from the food education diaries and consisted of six domains (Table 2). First, we calculated the total number of (1) food education sessions organized, (2) food-related topics covered, and (3) printed materials used. We also calculated the number of intervention periods during which at least one ECEC professional had (4) read at least one blog post from the study website and (5) followed at least one of the study’s social media channel (Facebook, Instagram, Twitter). In addition, we calculated (6) the number of different food education material types the groups had used during the intervention. The domain-specific cut-offs shown in Table 2 were used to score all the intervention-arm ECEC groups (*n* = 36) and a total DOI score was calculated as the sum of the domain-specific scores. The final score ranged from 0 to 16. As we were unable to link each individual participant with an ECEC group within an ECEC centre, we used the average DOI score of the groups for each ECEC centre in the analyses. The ECEC centres were grouped into high-intensity intervention (*n* = 5, DOI above median), low-intensity intervention (*n* = 6, DOI below median), and control group (*n* = 12, no intervention).

**Table 2:** Implementation of the food education intervention in the intervention arm and scoring criteria for the degree of implementation variable in the FoodStep study (2022)

Domain	Median	Interquartile range	Min–Max	Cut-offs	Scoring	Number of groups (%)
Food education sessions organized	13	16	0–46	0	0	1 (3)
				1–10	1	13 (36)
				11–20	2	11 (31)
				More than 20	3	11 (31)
Food-related topics covered	2	2	0–4	0	0	3 (8)
				1	1	10 (28)
				2	2	11 (31)
				3	3	6 (17)
				4	4	6 (17)
Printed materials used	1	1.25	0–4	0	0	6 (17)
				1	1	17 (47)
				2	2	4 (11)
				3	3	6 (17)
				4	4	3 (8)
Blog posts read ^1^	0	1	0–4	0	0	21 (58)
				1 or more	1	15 (42)
Social media channels followed ^1^	0	1	0–3	0	0	25 (69)
				1 or more	1	11 (31)
Food education material types used	6	5.25	0–12	0	0	1 (3)
				1–4	1	8 (22)
				5–8	2	14 (39)
				9 or more	3	13 (36)

^1^ Number of intervention periods during which at least one early childhood education and care professional had read at least one blog post from the study website / followed at least one social media channel (Facebook, Instagram, Twitter) of the study

### Participants

**Fig. 1:**
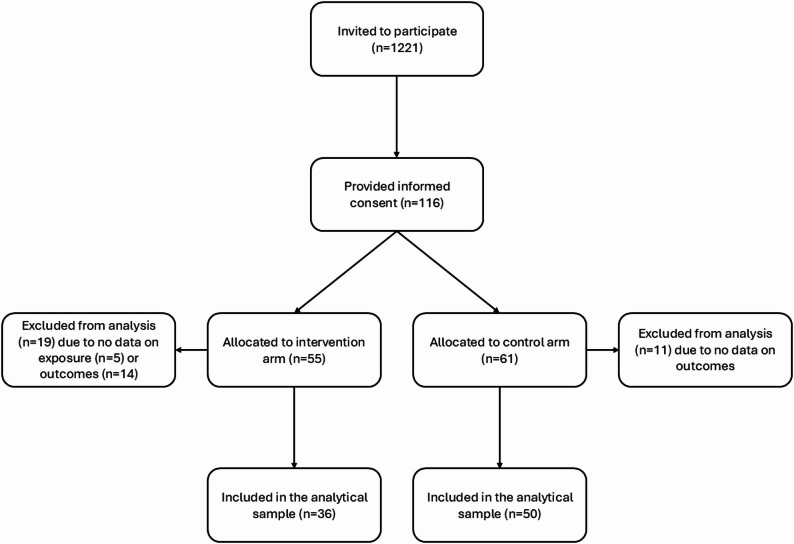
Flow of the participants in the FoodStep study (2022)

All children from the participating ECEC centres were invited to take part in a more detailed study collecting individual-level information between November 2021 and February 2022. Because of the COVID-19 protection measures, the parents of 3- to 5-year-olds were initially contacted by letter through the ECEC centres, but to encourage participation an additional face-to-face recruitment round was conducted as restrictions loosened in February 2022. Informed consent was obtained from 116 children (9.5% of those potentially invited), of which 111 provided background and/or dietary data (see Fig. 1 for participant flow diagram). The recruitment process is described in more detail in another manuscript [30]. A parent reported the age and sex of the child in the informed consent. Using an electronic questionnaire, they also reported the educational level of themselves and the other parent. The educational levels were categorized into three groups: low (upper secondary education or less), middle (bachelor’s degree or similar), and high (master’s degree or higher). In addition, they reported whether anyone in their family followed a vegetarian or vegan diet.

### Outcomes

This article reports three outcomes describing acceptance from different perspectives: acceptance, relative acceptance (acceptance divided by the number of foods served), and acceptance measured as food consumption. We report the three acceptance scores separately for vegetables, root vegetables, brassicas, legumes, berries, and fish, which were all targeted in the food education intervention. Summary scores for vegetables and legumes (vegetables, root vegetables, brassicas, and legumes), vegetables, legumes, and berries (the aforementioned + berries), and overall (all mentioned food groups) are also presented. The outcomes were assessed at baseline (before randomization) and at the end of the intervention (follow-up).

#### Acceptance and relative acceptance 

Through the electronic questionnaire, the parents reported how the child reacted when being offered certain food items. The answer options were 1=‘refused to touch food’, 2=‘touched food but did not put in/near mouth’, 3=‘put food to lips but not in mouth’, 4=‘put food in mouth but spat out / did not eat’, 5=‘ate food’, and 0=‘the child has not been offered this food in the last four weeks’. Earlier studies have used a similar questionnaire to assess children’s willingness to taste and food acceptability [33, 34]. Altogether, we selected 40 items that mirrored the food education topics in the intervention. These were (1) vegetables (12 items: tomato, cucumber, bell pepper, salad, rhubarb, mushrooms, aubergine, zucchini, leek, spinach, onion, garlic), (2) root vegetables (6 items: carrot, sweet potato, parsnip, beetroot, swede, radish), (3) brassicas (5 items: white cabbage, cauliflower, broccoli, Brussels sprout, kale), (4) legumes (6 items: pea, green bean, kidney bean, white bean, faba bean, chick pea), (5) berries (5 items: currant, bilberry, strawberry, lingonberry, raspberry), and (6) fish (6 items: perch, pike, whitefish, roach, herring, salmon).

For each participant, we calculated an acceptance score by summing the values obtained for vegetables (theoretical range 0–60), root vegetables (0–30), brassicas (0–25), legumes (0–30), berries (0–25), and fish (0–30) separately. We also calculated sum variables describing vegetable and legume (vegetables, root vegetables, brassicas, and legumes; 0–145), vegetable, legume, and berry (vegetables, root vegetables, brassicas, legumes, and berries; 0–170), and overall food acceptance (vegetables, root vegetables, brassicas, legumes, berries, and fish; 0–200). Based on the parent-reported information, we calculated the number of food items offered to the child during the past four weeks. This information was then used to create relative acceptance scores (range 0–5) by dividing the acceptance scores by the number of foods offered to the child. The calculations were performed separately for vegetables, root vegetables, brassicas, legumes, berries, and fish, and summary scores similar to acceptance scores were also calculated.

#### Food consumption 

The participating children’s food consumption was assessed using two separate electronic food frequency questionnaires (FFQs) filled in by parents (home FFQ) and ECEC personnel (ECEC FFQ). The home FFQ was developed by modifying an existing FFQ for pregnant Finnish women [35] and contained a total of 142 food rows, for which the parents reported their child’s average consumption outside ECEC time during the past month using answer options ‘rarely or never’, ‘1–3 times a month’, ‘1–2 times a week’, ‘3–4 times a week’, ‘5–6 times a week’, ‘1–2 times a day’, ‘3–4 times a day’, and ‘5 or more times a day’. A standard portion size was predefined for each FFQ row, and the parents were instructed to estimate consumption frequency as slightly higher or lower in cases where the child’s typical portion size was respectively larger or smaller than the predefined portion size.

For the development of the ECEC FFQ, we received information on the menus from the municipalities’ food services, which provide breakfast, lunch, and an afternoon snack for the children in full-time ECEC. For lunch meals, the ECEC FFQ included questions about the portion size of 27 main dish types (e.g. soup containing red meat) and eight types of side dish (e.g. white rice, whole-grain pasta, boiled potato). The frequency of consumption of each of these foods was derived from the menus obtained from the food services. For other food items (*n* = 79), there were questions related to both frequency of consumption and portion sizes. The ECEC FFQs were filled in by staff members of the ECEC centres and the instruction was to recall the child’s typical eating during the previous 1–2 months. The ECEC FFQ included pictures of dishes and food items to facilitate portion size estimation.

We calculated intakes of foods as g/d based on the daily frequencies and portion sizes. To disaggregate composite dishes into food items, we used averaged recipes from previous studies of Finnish 3- to 6-year-old children [36, 37] and the recipes given by the food services (ECEC FFQ). To take total energy intake into account, we calculated the total consumption of vegetables, root vegetables, brassicas, legumes, berries, and fish in g/MJ as the sum of the home and ECEC FFQs. To comply with the acceptance scores and the topics of the food education intervention, we also calculated summary variables to represent the intakes of vegetables and legumes (vegetables, root vegetables, brassicas, and legumes), vegetables, legumes, and berries (vegetables, root vegetables, brassicas, legumes, and berries), and all studied food groups (vegetables, root vegetables, brassicas, legumes, berries, and fish) in g/MJ.

### Statistical analysis

To examine the effectiveness of the food education intervention, we employed linear mixed models using acceptance, relative acceptance, and food consumption variables separately as outcomes. Sex (boy, girl), DOI (control, low DOI, high DOI), time point (baseline, follow-up), and the interaction between DOI and time point were included as fixed effects. ECEC centre and child ID were included in the models as random effects. Municipality was also tested as a random effect, but in most of the models, variance attributable to this was zero or close to zero. Excluding municipality did not affect the results. We present the estimates and 95% confidence intervals (CI) for all outcomes separately and consider the estimates for DOI × time-point interaction as representative of the effectiveness of the food education intervention. In addition, we used similar linear mixed models to investigate the associations between acceptance (both absolute and relative) and food consumption. These models included acceptance scores and sex as fixed effects and ECEC centre and child ID as random effects. All participants with data on DOI and at least one of the outcomes were included in the analytical sample (*n* = 86, 77% of those providing any data) and all participants with available data were included in each of the models (available case analysis). All analyses were performed using R (version 4.4.3, package ‘lme4’).

## Results

**Fig. 2:**
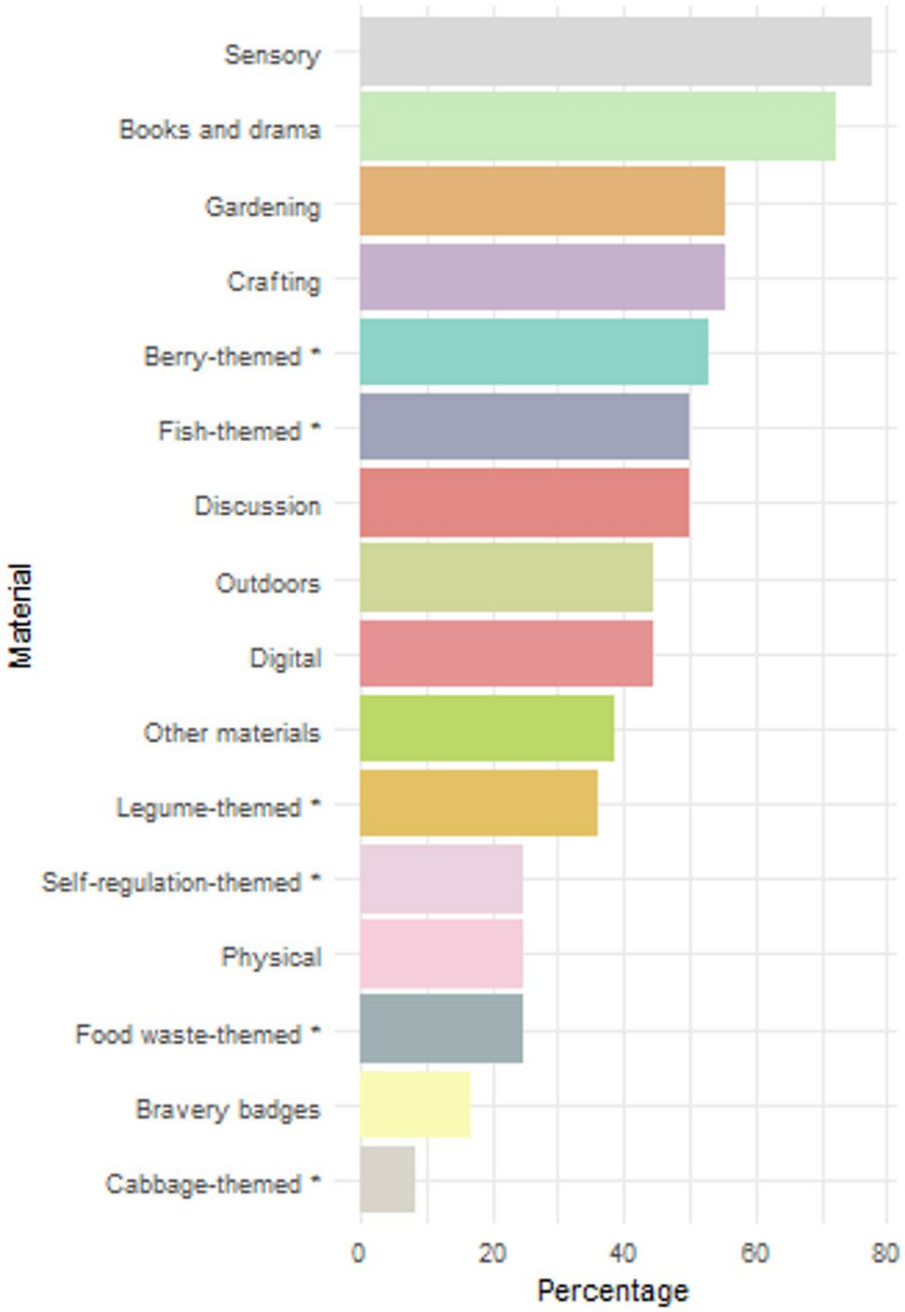
Use of food education materials in the ECEC groups within the intervention arm of the FoodStep study (2022). * specifically developed for the intervention by the research group

Altogether, 36 groups (62%) from the 11 intervention-arm ECEC centres reported how they had implemented food education during the intervention. Of them, a total of 13 groups (36%) had organized 1–10 food education sessions, while 11 groups (31%) reported 11–20 sessions and 11 groups (31%) more than 20 sessions during the 10-month intervention (Table 2). The average number of sessions was 16.1 (SD 11.4). The food-related topics of the intervention were covered as follows: legumes in 23 groups (64%), fish in 19 groups (53%), berries in 23 groups (64%), and brassicas in 9 groups (25%). Almost 80% of the groups reported using sensory food education materials (Fig. 2). Other commonly implemented food education activities were books and drama, gardening, and crafting. Of the materials produced by the FoodStep research group, berry- and fish-themed materials were also used in approximately 50% of the groups.

A total of 86 children were included in the individual-level analyses. Most of the children were from families with middle or high educational level (bachelor’s degree or higher). Of the participants, 50 (58%) were in the control-arm ECEC centres, whereas 36 (42%) were in the intervention-arm ECEC centres (Table 3). Of the intervention-arm participants, 15 (42%) were from high-intensity ECEC centres. All participants in the high-intensity ECEC centres were from the Päijät-Häme region. Vegetarian or vegan diet was followed in five families in the control arm.

**Table 3:** Descriptive characteristics of the participating children (*n* = 88) in the FoodStep study (2022) by degree of implementation

	Total		Control		Low DOI		High DOI	
	*n*	%	*n*	%	*n*	%	*n*	%
Sex								
Girl	47	54.7	29	58.0	12	57.1	6	40.0
Boy	39	45.3	21	42.0	9	42.9	9	60.0
Age								
3	38	44.2	21	42.0	8	38.1	9	60.0
4	27	31.4	17	34.0	7	33.3	3	20.0
5	21	24.4	12	24.0	6	28.6	3	20.0
Region								
Päijät-Häme	62	72.1	36	72.0	11	52.4	15	100.0
South Ostrobothnia	24	27.9	14	28.0	10	47.6	0	0.0
Vegetarian or vegan diet in the family								
No	74	86.0	43	86.0	19	90.5	12	80.0
Yes	5	5.8	5	10.0	0	0.0	0	0.0
Missing	7	8.1	2	4.0	2	9.5	3	20.0
Parental educational level								
Low	12	14.0	9	18.0	2	9.5	1	6.7
Middle	49	57.0	29	58.0	11	52.4	9	60.0
High	25	29.1	12	24.0	8	38.1	5	33.3

*DOI*  Degree of implementation

Compared with the control group, the children in the high-intensity intervention group showed higher acceptance of legumes at the follow-up, albeit the estimate was imprecise with wide confidence intervals (β = 4.27, 95% CI − 0.03 to 8.57, *p* = 0.06) (Table 4). This suggestive intervention effect corresponds roughly to a 140% increase in the acceptance score, whereas the acceptance score increased by 11% in the control group. A similarly potential, yet not statistically significant, intervention effect was also observed for fish (β = 4.04, 95% CI − 0.21 to 8.30, *p* = 0.07). The acceptability score for fish increased by 93% in the high-intensity intervention group, whereas it decreased by 13% in the control group. When considering how often the children had been offered the food in question, no statistically significant association for legumes or fish was observed. We found a potential, yet not statistically significant, intervention effect for relative acceptance of berries: the children in the high-intensity intervention group showed higher relative acceptance of berries at follow-up (β = 0.81, 95% CI − 0.01 to 1.62, *p* = 0.06), corresponding to approximately a 7% improvement in the high-intensity intervention group. At follow-up, children in the high-intensity intervention group consumed more legumes than children in the control group (β = 1.18, 95% CI 0.31 to 2.06, *p* = 0.01). Assuming a typical energy intake of 5.4 MJ/day, this intervention effect corresponds approximately to an increase of 11 g/day in legume consumption in the high-intensity intervention group when compared to the control group. The acceptance and relative acceptance scores were associated with food consumption in almost all food groups (Table 5).

**Table 4:** Intervention effects (degree of implementation × time-point interaction) in the FoodStep food education intervention (2022) with acceptance, relative acceptance, and food consumption as outcomes

	Outcome 1: Acceptance ^1^ (*n* = 75–86)				Outcome 2: Relative acceptance ^2^ (*n* = 75–86)				Outcome 3: Food consumption ^3^ (*n* = 76)			
	Estimate	Lower 95% CI	Upper 95% CI	*p* Value	Estimate	Lower 95% CI	Upper 95% CI	*p* Value	Estimate	Lower 95% CI	Upper 95% CI	*p* Value
Vegetables	3.52	−3.17	10.20	0.31	−0.19	−0.62	0.24	0.39	−1.70	−4.83	1.43	0.29
Root vegetables	2.85	−2.31	8.02	0.28	0.38	−0.26	1.02	0.25	−0.20	−2.71	2.32	0.88
Brassicas	2.11	−2.20	6.41	0.34	0.22	−0.78	1.22	0.67	−0.14	−0.77	0.49	0.67
Legumes	4.27	−0.03	8.57	0.06	0.71	−0.86	2.28	0.38	**1.18**	**0.31**	**2.06**	**0.01**
Berries	2.13	−3.42	7.68	0.46	0.81	−0.01	1.62	0.06	−0.04	−2.14	2.06	0.97
Fish	4.04	−0.21	8.30	0.07	0.07	−0.79	0.94	0.87	−0.43	−1.12	0.26	0.22
Vegetables + legumes ^4^	9.49	−7.50	26.47	0.28	−0.17	−0.61	0.26	0.44	−1.48	−6.86	3.91	0.59
Vegetables + legumes + berries ^5^	11.31	−9.59	32.21	0.29	−0.14	−0.51	0.24	0.48	−1.43	−7.51	4.65	0.65
Total	17.22	−7.21	41.65	0.17	−0.12	−0.48	0.24	0.52	−1.64	−7.04	3.77	0.56

Linear mixed models with sex, degree of implementation (DOI), time point, and the interaction between DOI and time point (intervention effect) as fixed effects and early childhood education and care centre and child ID as a random effects

Boldface indicates statistically significant estimates (p <0.05) along with their 95% confidence intervals

*CI*  Confidence interval

^1^ Theoretical range 0–30 for vegetables, 0–25 for root vegetables, 0–30 for legumes, 0–25 for berries, 0–145 for vegetables + legumes, 0–170 for vegetables + legumes + berries, and 0–200 for total

^2^ Theoretical range 0–5

^3^ In g/MJ

^4^ Vegetables, root vegetables, brassicas, and legumes summed together

^5^ Vegetables, root vegetables, brassicas, legumes, and berries summed together

**Table 5:** Associations between acceptance and relative acceptance scores with food consumption (g/MJ) in the FoodStep study (2022)

	Acceptance score ^1^ (*n* = 70–77)				Relative acceptance score ^2^ (*n* = 70–77)			
	Estimate	Lower 95% CI	Upper 95% CI	*p* Value	Estimate	Lower 95% CI	Upper 95% CI	*p* Value
Vegetables	0.07	−0.01	0.14	0.59	0.67	−0.73	1.97	0.32
Root vegetables	**0.08**	**0.01**	**0.15**	**0.03**	**0.66**	**0.06**	**1.27**	**0.03**
Brassicas	**0.05**	**0.03**	**0.07**	**< 0.001**	**0.19**	**0.12**	**0.27**	**< 0.001**
Legumes	**0.11**	**0.08**	**0.13**	**< 0.001**	**0.26**	**0.16**	**0.37**	**< 0.001**
Berries	**0.11**	**0.04**	**0.19**	**< 0.01**	0.17	−0.48	0.83	0.61
Fish	**0.05**	**0.03**	**0.07**	**< 0.001**	**0.25**	**0.16**	**0.34**	**< 0.001**
Vegetables + legumes ^1^	**0.10**	**0.06**	**0.15**	**< 0.001**	**2.69**	**0.72**	**4.59**	**< 0.01**
Vegetables + legumes + berries ^2^	**0.10**	**0.06**	**0.15**	**< 0.001**	**2.80**	**0.35**	**5.26**	**0.03**
Total	**0.07**	**0.04**	**0.11**	**< 0.001**	2.34	−0.05	4.65	0.05

Linear mixed models with sex as fixed effect and early childhood education and care centre and child ID as random effects

Boldface indicates statistically significant estimates (p < 0.05) along with their 95% confidence intervals

*CI*  Confidence interval

^1^ Theoretical range 0–30 for vegetables, 0–25 for root vegetables, 0–30 for legumes, 0–25 for berries, 0–145 for vegetables + legumes, 0–170 for vegetables + legumes + berries, and 0–200 for total

^2^ Theoretical range 0–5

^3^ Vegetables, root vegetables, brassicas, and legumes summed together

^4^ Vegetables, root vegetables, brassicas, legumes, and berries summed together

## Discussion

This study suggests that food education to promote a sustainable and healthy diet can be embedded in pedagogic routines within the ECEC environment. Moreover, it showed that intensively implemented food education in ECEC centres can increase legume acceptance and consumption in children. To the best of our knowledge, this is the first partially randomized controlled trial to modify legume supply in the ECEC menu coupled with food education activities. Legumes are under-consumed in this age group and increasing their consumption is beneficial for both health and, when replacing meat, the environment, making it a priority to design and pilot effective interventions.

Evaluating the feasibility of the food education intervention is challenging. Only 31% of the groups within the intervention-arm ECEC centres reported organizing more than 20 food education sessions during the 10-month intervention period, suggesting somewhat compromised feasibility. Additionally, the ECEC centres in which the intervention was implemented more intensively were from the Päijät-Häme region, which is a more urban area compared with the South Ostrobothnia region. This indicates that there may be regional differences in feasibility, possibly affected by multiple factors, such as the number of ECEC centres, the food service provider’s ability and willingness to cooperate in food education, the perceived importance of food education, and attitudes towards environmental sustainability issues and health within the surrounding society. However, based on the feedback from the ECEC centres, it is very likely that reporting on the food education sessions was more of a barrier than organizing them. Thus, it is possible that food education was under-reported in some if not all ECEC centres. For future studies, we recommend developing less burdensome yet sufficiently accurate methods for assessing implemented food education.

The most commonly implemented food education activities in the current study were sensory food education, books and drama, gardening, and crafting. These activities were fairly similar to those reported in earlier studies (see, for example [12, 16, 20, 28]), , suggesting wide acceptance and willingness to implement by ECEC professionals. To enhance food education, ECEC professionals should have access to evidence-based, high-quality materials that focus specifically on sustainable foods, such as legumes, which have not traditionally been the focus of food education. Future studies should explore which food education materials and activities are most effective in promoting sustainable diets among children.

This study demonstrated that the children in the high-intensity intervention group consumed more legumes compared with the children in the control group, providing support for earlier studies with similar findings [26, 28]. Assuming an age-appropriate typical energy intake of around 1300 kcal, the observed increase is equivalent to approximately 11 g/d, which would triple the current average consumption of legumes in this age group [6]. Unlike earlier studies, the food education intervention did not seem to increase the acceptance or consumption of other foods, such as vegetables and berries (see, for example [12, 16, 22]), . A likely explanation is that there is probably more room for improvement in legume consumption, whereas the consumption of vegetables and fruit is already much closer to target levels [6]. Moreover, of the food groups targeted in the menu modifications, legumes was the one for which the relative amounts changed the most [32], providing opportunities for the children to consume more legumes in ECEC.

Interestingly, we saw a more noticeable intervention effect on legume consumption than legume acceptance. As acceptance was assessed using a five-point scale ranging from ‘refused to touch food’ to ‘ate food’, it could be hypothesized that compared with food consumption, the acceptance score would be more sensitive to the effects of legume-themed interventions. However, legumes served in ECEC were mostly ingredients in dishes, so not all children necessarily knew they were eating them. This might have shown in increased consumption. Moreover, as acceptance was parent reported, it can be considered a parent’s subjective perception of their child’s food-related behaviour, whereas the increase in consumption was likely driven by the increased amounts of legumes served in ECEC. Research has shown that social, biological, economic, and psychological factors are associated with parental perception of their child’s diet [38] suggesting that parents may be unable to reliably report food acceptance for their children. Thus, food consumption, although also relying on self-reporting, may reflect food acceptance more objectively, particularly in this study where food consumption was reported both by parents and ECEC personnel.

In Finland, children in full-time ECEC are served breakfast, a hot meal (lunch), and a snack daily [31]. Thus, the ECEC setting not only plays a considerable role in their diet but also holds significant potential for sustainability transformation. By replacing animal-based foods with plant-based foods, municipal food services preparing meals for ECEC centres can reduce the climate impact of their menus [39, 40]. Importantly, these modifications can be made in a way that does not increase food waste [39, 41] and reduces costs [39], showing that the environmental sustainability of foods served in ECEC can be improved without compromising economic sustainability. The adults in the ECEC centres play a pivotal role as their opinions and practices as role models are associated with the children’s diets [42]. Legume consumption in Finland is minimal also within the adult population [43], which makes it challenging for ECEC professionals to act as role models. Education on sustainable food should be systematically embedded in the training of ECEC professionals and teachers to ensure that they acknowledge the potential of building a positive narrative around plant-based diets, food, and health.

Shifting towards plant-based diets in ECEC should not come at the expense of acceptability. While this study showed that food education can increase legume consumption, repeated taste exposure is generally considered the most effective intervention strategy to increase vegetable consumption in young children [44]. As some legumes have a challenging taste profile, effective food education practices in ECEC settings are needed to familiarize children with legumes. To support food education, food services need to continuously develop their plant-based recipes to ensure that children get the opportunity to try new foods and dishes repeatedly. The successful introduction of plant-based foods on menus contributes to maintaining the social sustainability of food provision in ECEC and can be supported by measures such as a gradual increase in plant-based dishes, training of food service and ECEC personnel, and the engagement of different interest-holders in the process [45].

This study has several strengths that increase the reliability of the obtained results. First, we employed a partially cluster-randomized setting, which is more rigorous than commonly used quasi-experimental designs and can thus provide an indication of causal relationships. Second, we assessed food acceptance using three measures: parent-perceived acceptance, relative acceptance that considers foods offered to the child, and food consumption reported separately by parents and ECEC professionals. The obtained results can be considered rigorous and reliable because they remained fairly similar regardless of the food acceptance outcome inspected. We also found statistically significant associations between the outcomes for most of the food groups, supporting the argumentation. Third, relying mostly on parent-reported data on all the outcomes gives credibility to the results, as the parents were unaware how intensely the food education intervention was implemented in the ECEC centres.

Some limitations must be considered when evaluating the reliability of the results. For example, for practical reasons, we were able to randomize ECEC centres only in one of the four participating municipalities. It is thus possible that the ECEC centres assigned to the intervention arm were different from those in the control arm. However, most of the participating children (73%) were from the municipality where randomization was used, decreasing the possibility of systematic error. Moreover, we were unable to conduct a priori power calculations for this partially cluster-randomized intervention. Effect size estimates for the preregistered primary outcomes (ECEC food supply, climate impact, and food service costs) were unavailable from the literature, and intra-cluster correlation coefficients had not been established from comparable settings. It is thus likely that the study was underpowered, which probably contributed to some of the findings not reaching statistical significance. Given the low participation rate and challenges in randomization, these novel findings require replication in larger samples before definitive conclusions about effectiveness, scalability, and wider implementation can be drawn. The questionnaires used to assess the outcomes (acceptance, relative acceptance, and food consumption) were not validated, which reduces the reliability of the results. However, converging results suggest at least somewhat acceptable construct validity. Additionally, food education activities were not reported by all of the ECEC groups within the 11 intervention-arm ECEC centres. In addition, we had no information about which ECEC group within the intervention ECEC centres each participating child was from. Thus, instead of using group-specific DOI scores, we had to calculate a mean DOI score for each of the intervention-arm ECEC centres, which decreased variability and could have led to attenuation in the observed associations. Finally, the participating children were mostly from highly educated families. As individuals with higher education and more favourable health behaviours are more likely to take part in studies [46], it is possible that the current study sample showed less variance in food consumption, leading to attenuated associations.

## Conclusions

This study highlights the feasibility and potential effectiveness of structured, ECEC-delivered food education interventions in increasing children’s consumption of legumes, which generally have a smaller environmental impact compared with many animal-based foods. It supports integrating legumes and other environmentally sustainable foods into both food provision and food education activities. For the intervention to be feasible, ECEC professionals must have access to attractive and rapidly deployable food education materials and activities. Sufficient resources for planning and implementing food education are also needed. However, even the most intensive food education is not enough to increase acceptance and consumption of environmentally sustainable foods – repeated taste exposure is always necessary. Thus, collaboration between interest-holders at national, municipal, food service provider, and ECEC levels is needed to ensure that children are provided with environmentally sustainable and healthy food. 

## Data Availability

Researchers interested in the data from this study may contact principal investigator Suvi M. Virtanen, suvi.virtanen@thl.fi.

## Abbreviations

CI: Confidence interval
DOI: Degree of implementation
ECEC: Early childhood education and care
FFQ: Food frequency questionnaire
